# A multidisciplinary approach to eosinophilic esophagitis presenting with feeding dysfunction

**DOI:** 10.1093/omcr/omaf096

**Published:** 2025-07-14

**Authors:** Emily Sitner-Medvedovsky, Nina Gabriella Sparacio, Bibiana Bernal, Ankita Prasad, Hillary Polk, Oksana Nulman, Shuchi Jani, Cathy Fan, Brande Brown, Kenny Castro Ochoa

**Affiliations:** Department of Pediatrics, New York Presbyterian Brooklyn Methodist Hospital, 506 6th Street, Brooklyn, NY 11215, New York; Department of Pediatrics, New York Presbyterian Brooklyn Methodist Hospital, 506 6th Street, Brooklyn, NY 11215, New York; Department of Pediatrics, New York Presbyterian Brooklyn Methodist Hospital, 506 6th Street, Brooklyn, NY 11215, New York; Department of Pediatrics, New York Presbyterian Brooklyn Methodist Hospital, 506 6th Street, Brooklyn, NY 11215, New York; Department of Pediatrics, New York Presbyterian Brooklyn Methodist Hospital, 506 6th Street, Brooklyn, NY 11215, New York; Department of Pediatrics, New York Presbyterian Brooklyn Methodist Hospital, 506 6th Street, Brooklyn, NY 11215, New York; Department of Pediatrics, New York Presbyterian Brooklyn Methodist Hospital, 506 6th Street, Brooklyn, NY 11215, New York; Department of Pathology and Laboratory Medicine, Division of Gastroenterology, Pancreas, and Liver, Weill Cornell Medicine 525 East 68th St, NY 10021, New York; Department of Pediatrics, New York Presbyterian Brooklyn Methodist Hospital, 506 6th Street, Brooklyn, NY 11215, New York; Department of Pediatrics, New York Presbyterian Brooklyn Methodist Hospital, 506 6th Street, Brooklyn, NY 11215, New York; Division of Pediatric Gastroenterology and Nutrition, Weil Cornell Medicine 525 East 68th St, NY 10021, New York

**Keywords:** eosinophilic esophagitis, feeding difficulties, EoE, food hypersensitivity, multidisciplinary health team, topical steroids, elemental diet

## Abstract

Eosinophilic Esophagitis (EoE) is a chronic, immune-mediated disorder characterized by eosinophilic infiltration of the esophageal mucosa. EoE presents a clinical challenge as the underlying esophageal dysfunction results in diverse symptomatology, ranging from feeding difficulties and failure to thrive in infants to dysphagia and food impaction in older children. The treatment and long-term management of EoE involves a multidisciplinary approach that may include topical glucocorticosteroids, diet therapy and other evolving pharmacotherapies. We present a case of a 12-month-old previously healthy female with a 3-week history of illness followed by an acute presentation of feeding intolerance and hypersensitivity reactions who was subsequently diagnosed with EoE.

## Introduction

Eosinophilic Esophagitis (EoE) is a chronic, immune-mediated disorder characterized by eosinophilic infiltration of the esophageal mucosa. The etiology of EoE is complex, likely involving both a genetic predisposition and environmental triggers. Recent studies have implicated polymorphisms in genes associated with epithelial barrier function and immune regulation, while allergen exposure and dysregulated immune responses to dietary antigens have also been identified as potential triggers for the inflammatory cascade seen in EoE [1]. A recent meta-analysis reported a global incidence of 5.1 cases and a prevalence of 19.1 cases per 100 000 children[2]. The disease prevalence is highest in those children who suffer from food impaction or dysphagia (63–88%) [3].

The diagnosis of EoE relies on a combination of clinical, endoscopic, and histological findings. Upper endoscopy with esophageal biopsy remains the gold standard for diagnosis, with characteristic findings including esophageal rings, linear furrows, white exudates, and esophageal narrowing/strictures.The management of EoE in the pediatric population is involves a combination of dietary modifications, pharmacotherapy, and endoscopic interventions. Elimination diets, such as the six-food elimination diet (SFED) or the elemental diet, have been shown to induce remission in particularly those with identified food triggers [4]. Pharmacologic therapies, including proton pump inhibitors (PPIs), topical corticosteroids, and biologic agents targeting specific cytokines involved in the inflammatory cascade have also shown efficacy in symptom control and histologic improvement [3].

## Case presentation

A 12-month-old female with a history of eczema and a family history of atopy presented with three weeks of non-bilious, non-bloody vomiting which started upon introduction of cow’s milk at 11 months of age with associated weight loss during the same period. She was transitioned back to formula feeding and later to elemental feeds without significant improvement. On admission her vital signs were normal without signs of dehydration on exam but had generalized xerosis and scaly red rashes on the trunk and back.Three weeks prior to presentation, she had roseola and otitis media and was prescribed amoxicillin which caused angioedema, treated with diphenhydramine. She had previous allergic episodes characterized by angioedema while eating sweet potatoes at six months of age and another episode with hives and difficulty swallowing that was thought to be a result of her throat closing-requiring Epinephrine.

**Figure 1:**
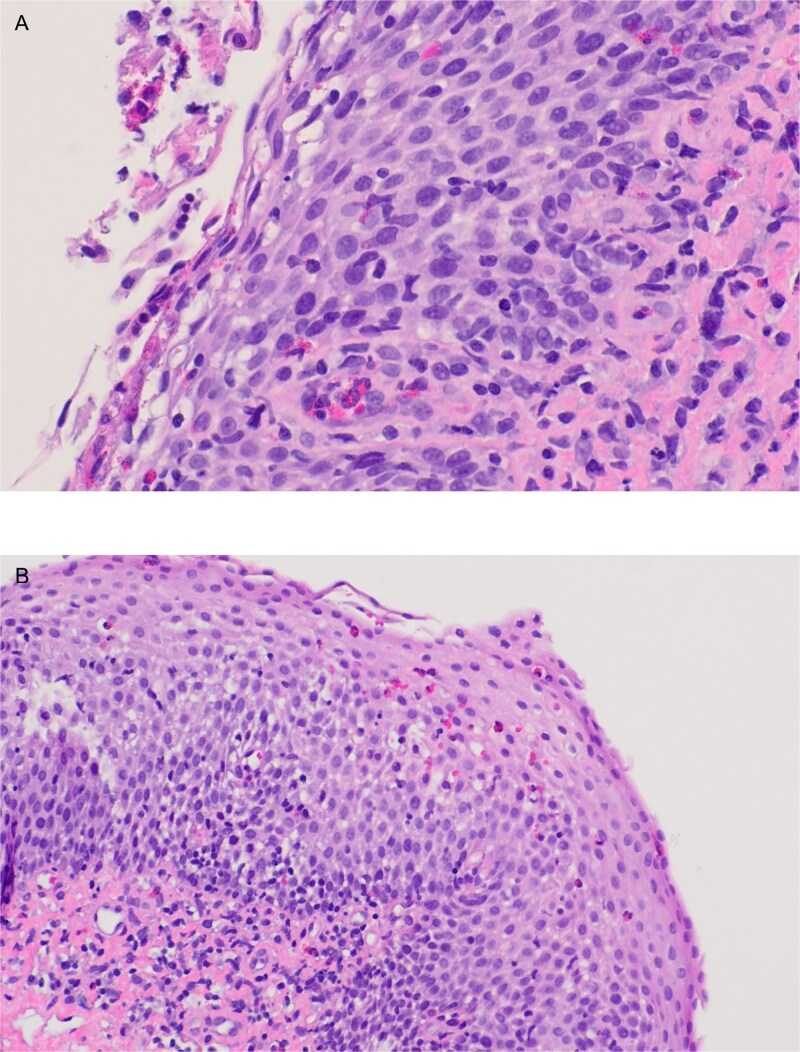
Active esophagitis with numerous eosinophils, superficial eosinophilic microabscesses, and degranulating eosinophils in scale crust throughout the upper, mid, and distal.

**Figure 2:**
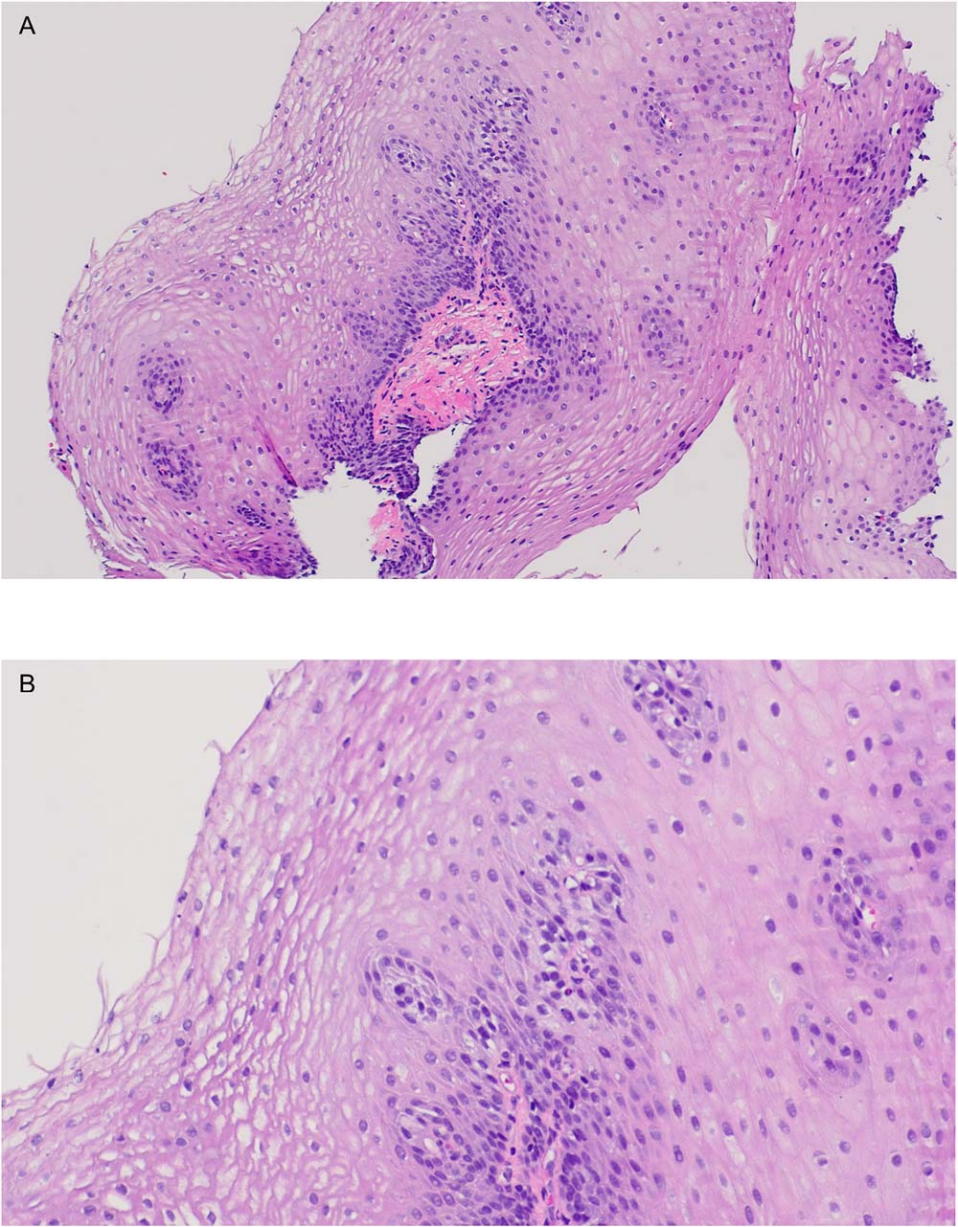
Squamous esophageal epithelium without evidence of intraepithelial eosinophils after budesonide and elemental diet.

During her hospital stay she experienced two episodes of anaphylaxis to unknown triggers, responding to epinephrine administration. Initial laboratory tests revealed eosinophilia and elevated IgE, TTG IgG, and gliadin peptide IgG antibodies. Based on her history of eczema, food allergies, and associated food intolerance we suspected EoE. She was initiated on lansoprazole and nasogastric feeds of lactose and soy oil-free elemental diet. An esophagogastroduodenoscopy with biopsy was performed showing esophageal mucosal with longitudinal furrows, white plaques, and mucosal friability in the mid and distal esophagus. On pathology, active esophagitis with esophageal eosinophilic infiltration from 3 separate portions of the esophagus, with the highest of 35 per HPF in the distal esophagus, as well as microabscesses throughout the upper, mid, and distal esophagus, were noted, consistent with the diagnosis of EoE (Fig. 1A and B). She was started on swallowed viscous budesonide with significant improvement in symptoms and was later discharged with a course of lansoprazole, viscous steroids, and an elimination diet. A follow-up endoscopy with biopsy was completed 3 months later which showed a normal esophagus and no eosinophils on the pathology report (Fig. 2A and B).

## Discussion

Fifty-three percent of patients who were diagnosed with EoE were found to have an association with asthma, allergic rhinitis, or eczema while forty-three percent had a first-degree relative with asthma or food allergies, as seen in our patient [5]. Infants and toddlers are more likely to present with failure to thrive and feeding difficulties [5]. To establish the diagnosis, endoscopic evaluation is the gold standard and it is essential to have more than 15 eosinophils per high power field in at least one of the biopsy sites [2].

It was thought that the unremitting emesis in our patient was related to her unresolved illness (roseola infection complicated by otitis media); however, due to its persistence in the setting of weight loss, further work-up was required. *Liacouras et al* demonstrated that dysphagia and reflux symptoms were the most common presenting complaints; 70% presented with vomiting or regurgitation. Vomiting is a non-specific symptom that can be associated with a range of illnesses and can be mistaken for food-induced protein enterocolitis or IgE-mediated food allergies if it appears food-dependent [6]. The additional complexity to this case was that the patient had been eating solids for several months at presentation, and the onset of persistent vomiting was subacute (three weeks prior). During hospital admission, the patient did not exhibit any gagging or choking behaviors, often suggestive of dysphagia, nor any associated pain or food aversion symptoms that may be present in older children. A study done in the North Denmark region in 2022 found the average age of EoE diagnosis was four years and six months and recognized that there is often a delay in diagnosis due to non-specific symptom presentation [7].

Dietary elimination, mostly with an amino acid-based formula (elemental) diet has proven successful, as noted in our patient once EoE was confirmed (Neocate Junior®). Dietary changes with removal of known or potential triggers, or empiric by eliminating the most common known food allergens (nuts, shellfish, wheat, diary, soy, eggs) can be also be targeted [7]. Since patients who show partial improvement in their eosinophil count may report a higher resposne rate than their histology would suggest, the histological status and symptoms do not correlate well in EoE. This often results in undertreatment and increased risk of stricture formation; therefore, histological remission is generall considerend to be better than symptomatic improvement [8].

Recent advances in the management of EoE involve targeted therapy. Dupilumab is an IgG4 human monoclonal antibody that binds to IL-4Ra and blocks the shared receptor component for IL-4 and IL-13, key central drivers of type 2 inflammation. Dupilumab is the only drug that the FDA has approved for treatmeent of EoE for adults and children over the age of 12 [9]. It can also be used as a first-line agent in individuals with several coexisting atopic disorders including chronic sinusitis with nasal polyps, atopic dermatitis, and moderate to severe asthma, or in individuals who would strongly prefer not to use topical ingested steroids or dietary restrictions. Dupilumab can be considered as a step-up therapy for substantial weight loss, poor growth, frequent esophageal dilatation, or in cases of severe diet restrictions.

## Conclusion

We emphasize a noteworthy presentation of EoE in a 12-month-old previously healthy female with acute feeding intolerance and weight loss. Interestingly, she presented with persistent vomiting and hypersensitivity reactions to food that she had previously tolerated. Additional diagnostic challenges included the patient’s non-specific symptomatology and the lack of food aversion or anorexia despite weight loss. This case report highlights the potential for anchoring bias and the significance of maintaining a wide differential diagnosis, even for seemingly simple cases. As the diagnostic awareness of the clinician evolves in conjunction with the increasing incidence and prevalence of pediatric EoE, we stress that its treatment and management manifest as an up-and-coming area of investigation.

### Informed Consent

Informed consent was obtained by the parent of the patient.
